# Wild and Micropropagated *Artemisia eriantha* Infusions: In Vitro Digestion Effects on Phenolic Pattern and Antioxidant Activity

**DOI:** 10.3390/plants13010085

**Published:** 2023-12-27

**Authors:** Rachele Rocchi, Marika Pellegrini, Paola Pittia, Loretta Pace

**Affiliations:** 1 Istituto Zooprofilattico Sperimentale dell’Abruzzo e del Molise “G. Caporale”, Via Campo Boario, 64100 Teramo, Italy; r.rocchi@izs.it; 2 Department of Life, Health and Environmental Sciences, University of L’Aquila, Via Vetoio, 67100 L’Aquila, Italy; marika.pellegrini@univaq.it (M.P.); loretta.pace@univaq.it (L.P.); 3 Faculty of Bioscience and Technologies for Food, Agriculture and Environment, University of Teramo, Via Balzarini, 1, 64100 Teramo, Italy

**Keywords:** Apennines Genepì, conservation, phytochemistry, plant science, GID, polyphenols, antioxidants, HPLC-DAD

## Abstract

This study investigated the in vitro simulated gastrointestinal digestion (GID) effects on wild and micropropagated Apennines Genepì infusions. Wild and micropropagated infusions were compared for their antioxidant activity, phenolic contents, and polyphenolic profiles before and after GID. Before digestion, the wild infusions had higher amounts of phenolic compounds and antioxidant activity than the micropropagated ones. Instead, after digestion, the differences in the total phenolic content (TPC) and antioxidant activity between wild and micropropagated infusions were less pronounced. The changes in the TPC and phenolic profiles revealed the presence of several chemical transformations and rearrangements that resulted in compounds with different reactivity and antioxidant potential. Without enzyme actions, the wild infusion digest undergoes higher modifications than those obtained from the micropropagated ones. The current study offers the first concrete proof of the impact of GID on the polyphenolic chemicals present in infusions of wild and micropropagated Apennines Genepì and their antioxidant properties. Our findings are essential for future in-depth analyses of Apennine Genepì infusions and their potential impacts on human health.

## 1. Introduction

*Artemisia* is an Asteraceae family’s genus comprising several species broadly distributed worldwide and with important economical and phytotherapeutic significance [1,2,3,4,5]. The vast ecological plasticity of this genus allows plants to occur in the most diverse environments (e.g., arid zones, mountains, sea, and wetlands) to be naturalised in several environmental conditions [6]. Several investigations have been carried out to preserve this plant, and some actions have been promoted. In the Abruzzo region located in the center of Italy, a dedicated project allowed clones obtained from micropropagated plants to be cultivated for the conservation of the endangered species in the Campo Imperatore botanical garden of the University of L’Aquila (Gran Sasso Monti della Laga National Park, Abruzzo Region, Italy). The micropropagation technique can be applied on a large scale for the commercial production of clones [7]. In return, the establishment of commercial clones and their valorisation could contribute to limiting the illegal and undiscerning picking up that threatens this species.

Plants belonging to the *Artemisia* genus are usually used as bittering agents in traditional and commercial alcoholic beverages [8]. Among the latter, Genepì liqueur, obtained by the infusion of the aerial parts of the plant in ethanol, is renowned in folklore medicine for its thermogenic properties and recommended to counteract airway infections, weakness, and indigestion [9]. It is diffused in many Italian regions, and in the Alps areas (North Italian regions), Genepì liqueur is obtained from *Artemisia genipi* Weber, *Artemisia umbelliformis* Lam., and *Artemisia glacialis* L. [10], while, in the Apennines (Abruzzo region), a liqueur with the same name, along with other infusions, are made from the species *Artemisia eriantha* [11]. Besides the characteristic sensory properties and bitterness, the main interest in the liqueurs and infusions is related to the beneficial effects of the extracts on health and diverse bioactive properties. The scientific literature reports evidence of *Artemisia* species bioactive compounds and their antihypertensive [12] antitumoral [13], anti-inflammatory [14], hepatoprotective [15], hypoglycaemic [16], hypolipidemic [17], and antioxidant properties [18,19]. The biological properties ascribed to this species are mainly related to thujones, terpenoid ketones that can be found principally in the small, yellow flower heads but, also, in lower concentrations, leaf apices [20], and phenolic compounds [7]. The concentrations of these bioactive compounds are a key element in their effects on human health. If exceeding the acceptable daily intake of 5.0 mg/person for 2 weeks, thujones have a neurotoxic behaviour [21]. Their content in *A. eriantha* does not increase with micropropagation. Previous studies have shown that micropropagated populations have lower thujones and higher security than wild populations [22]. In other cases, similar concentrations have been found between the two populations [11].

The beneficial health effects of a natural product are associated with the presence and concentration of the specific micronutrients and secondary metabolites [23]. High temperatures, changes in pH, and the presence of other molecules could induce changes in the pH or ionic force, cause degradation reactions, or trigger the formation of other moieties with different bioactivity evidenced both in vitro and in vivo [24]. Bioactive compounds’ availability for absorption in the gut may vary significantly in the same food depending on these intrinsic matrix factors and processing [25]. Several scientific works have highlighted the significant effect of processing and storage conditions and those occurring during digestion on the health benefits of bioactive molecules. Bioaccessibility and bioavailability are not synonymous terms [26]. In particular, “bioaccessibility” corresponds to the amount of an ingested nutrient or food compound released from the food matrix and potentially available for absorption in the gut after digestion. At the same time, “bioavailability” refers to the amount of an ingested nutrient available after digestion that the body can use through an absorption mechanism for utilization in normal physiological functions and metabolic processes [27,28,29]. The experimental procedures to determine the bioaccessibility and bioavailability can involve both human (in vivo) or simulated studies (in vitro) performed in the laboratory [28,29]. The current standardised method commonly utilised to assess bioactive compounds’ stability is in vitro simulated gastrointestinal digestion (GID), which allows for obtaining results without animal models and with limited time, costs, and variables [30].

The influence of the GID on the antioxidant activity of *Artemisia gorgonum* Webb infusion [31] and *Artemisia lactiflora* dried powder and fresh extracts [32] has been recently studied. Still, more information is needed on the modifications of the polyphenolic profile and antioxidant activity of *A. eriantha* during gastrointestinal digestion and the possible contribution of environmental factors that could affect these compounds’ bioaccessibility. Given the wide use in folk pharmacopeia, we hypothesised interesting phenolic profiles and antioxidant activities after GID of the infusions. Thus, this work aims to evaluate the effect of in vitro GID on the phenolic profile and antioxidant activity of Genepì infusions from Apennines Genepì aerial parts. Agamic propagation of this rare entity, obtained by in vitro technique, is necessary for its protection as required by an Italian regional law (L.R. n.47, 11 September 1979) and the European Habitats Directives 92/43/EEC, Annex V [33]. The ultimate purpose of the study is to promote a technique that allows the conservation of the species and possible commercial exploitation without impacting natural populations. Previous research has demonstrated that micropropagated Genepì has several potential commercial applications (e.g., essential oils, bioactive compounds, and the low presence of toxic thujones) [7,20]. The differences between wild and micropropagated populations, before and after GID, were also investigated to evaluate the suitability of commercial clones as valid alternatives to natural plants.

## 2. Results

### 2.1. Polyphenolic Profiles

**Figure 1:**
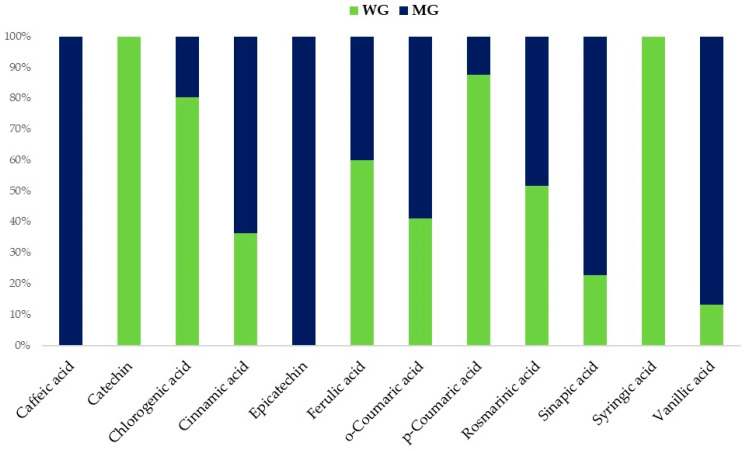
Stacked column chart of the phenolic compounds (%) of wild (WG) and micropropagated (MG) infusions.

In Figure 1, the polyphenolic profiles of the wild and micropropagated Genepì (WG and MG, respectively) infusions before digestion are presented.

Wild and micropropagated infusions are characterised by the presence of phenolics belonging to different classes (e.g., benzoic acids, cinnamic acids, flavan-3-ols, and flavonols). WG infusions showed a higher total content of the polyphenolic compounds (*p* < 0.001) than the MG (1531 mg 100 g^−1^ dry weight (DW) vs. 767 mg 100 g^−1^ DW), and its pattern was dominated by the high presence of *p*-coumaric (ca. 50% of the total phenolics content), followed by chlorogenic acid and catechin. Syringic acid and catechin were detected only in the WG infusion. On the contrary, two exclusive phenolics distinguished the MG infusion, i.e., caffeic acid and epicatechin. By comparing the two patterns, the highest concentrations of cinnamic (*p* < 0.001), *o*-coumaric (*p* < 0.01), sinapic (*p* < 0.01), and vanillic acids (*p* < 0.001) were found in MG. At the same time, WG presented the highest concentrations of ferulic (*p* < 0.01), chlorogenic (*p* < 0.001), and *p*-coumaric acids (*p* < 0.001). No significant differences (*p* > 0.05) between MG and WG were found for rosmarinic acid.

The effects of the GID on the WG and MG polyphenolic profiles are reported in Table 1. In WG infusions, the digestion caused a significant decrease of *p*-coumaric acid (ca.—95%). Conversely, high increases of chlorogenic, sinapic, and *o*-coumaric acids and the appearance of new compounds, i.e., chicoric and caffeic acids and rutin, were found. A similar trend to the WG one was observed by MG infusions after digestion that, except for the decrease of sinapic acid, showed the complete absence of *p*-coumaric acid and the presence of quercetin, kaempferol, and chicoric acids. The data of the control samples obtained by applying the environmental conditions of in vitro digestion without enzymes (i.e., pH, ionic force, and temperature) are also reported in Table 1. The results show the role of the physical–chemical conditions of the system (without enzymes) on the disappearance or appearance of phenolic compounds. Generally, the decreases and increases recorded for individual compounds were stronger in the presence of digestion than the control. For WG, the exceptions included cinnamic acid, *o*-coumaric acid, and rosmarinic acid, for which similar changes were recorded in the control and digested samples compared to the undigested sample. For MG, a similar trend was recorded for caffeic acid, *o*-coumaric acid, rosmarinic acid, and the total concentration.

**Figure 2:**
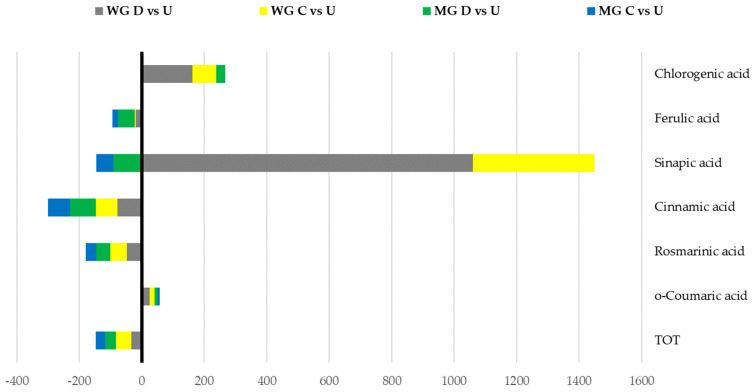
Stacked column chart of the variation percentages recorded for phenolic compounds of the wild (WG) and micropropagated (MG) infusions. D, digested; U, undigested; C, control (digestion without enzyme). On the left-hand side of the axis (−400–0%), the decreases in phenolic compounds in the stacked columns are presented. On the right-hand side (0–1600%), increases in the stacked columns are presented.

As presented in Figure 2, regarding the phenolics already present in the undigested samples, the percentages of the variations were similar between WG and MG for the total phenols and *o*-coumaric, rosmarinic, cinnamic, ferulic, and chlorogenic acids. For synapic acid, both in the presence and absence of enzymes, the MG samples showed a decrease, while the WG showed a strong increase.

### 2.2. Antioxidant Activity

The results of the total phenolic content (TPC) and antioxidant activity (DPPH, 2,2-diphenyl-1-picrylhydrazyl; ABTS, 2,2′-azino-bis(3-ethylbenzothiazoline-6-sulphonic acid); FRAP, ferric-reducing antioxidant power) analyses of both the undigested and digested WG and MG infusions are reported in Table 2.

The undigested WG infusions showed a higher TPC content than the MG ones (*p* < 0.01), and a similar behaviour was found for the results of all the antioxidants assays (*p* < 0.01). The results of the ABTS and DPPH antioxidant assays showed a positive correlation with the TPC, while a negative one was found for FRAP.

The in vitro digestion induced a significant decrease of the total content of the phenolic compounds of both infusions but to a different extent, depending on the initial infusion composition, the test method, and the digested samples still presented.

In general, independently from the mechanisms of action of the methods used for antioxidant activity determination and the corresponding results, the reduction of the bioactivity was higher in WG than MG. However, despite the different initial phenolic profiles and changes induced by the in vitro digestion in the phenolics’ composition, the results of the antioxidant activity of the digested WG and MG infusions were similar, with no significant differences between them (*p* > 0.05).

**Figure 3:**
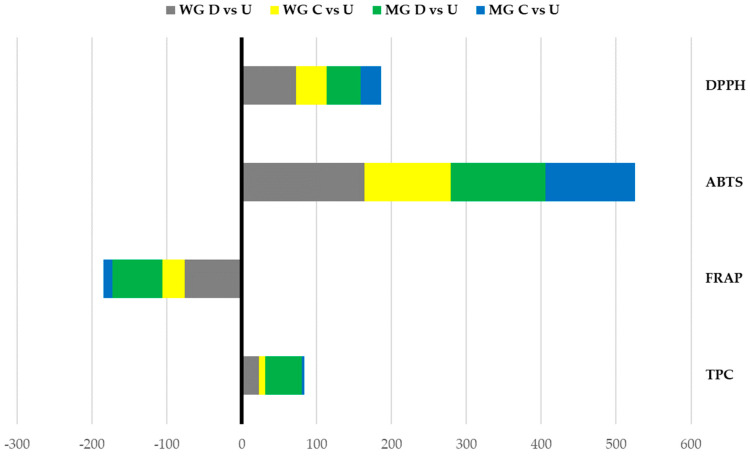
Total phenolic content (TPC) and antioxidant activity (FRAP, ABTS, and DPPH) variation percentages of undigested (U), control (C, without enzymes), and digested (D) infusions of wild (WG) and micropropagated Genepì (MP) infusions. On the left-hand side of the axis (−300–0%), the decreases in antioxidant activity in the stacked columns are presented. On the right-hand side (0–600%), increases in the stacked columns are presented.

As showed for the HPLC-DAD results, in both the TPC and antioxidant activity evaluated by the different methods, the digestion carried out without enzymes determined changes in the phenolic contents and antioxidant activities of the infusions (Figure 3). In both MG and WG, environmental conditions during the digestion process (e.g., pH and temperature changes) contributed to the degradation and rearrangements of the polyphenolic profiles and antioxidant activities. Generally, these changes were significantly lower than the ones recorded for digestion (*p* < 0.05).

### 2.3. Statistical Analysis

**Figure 4:**
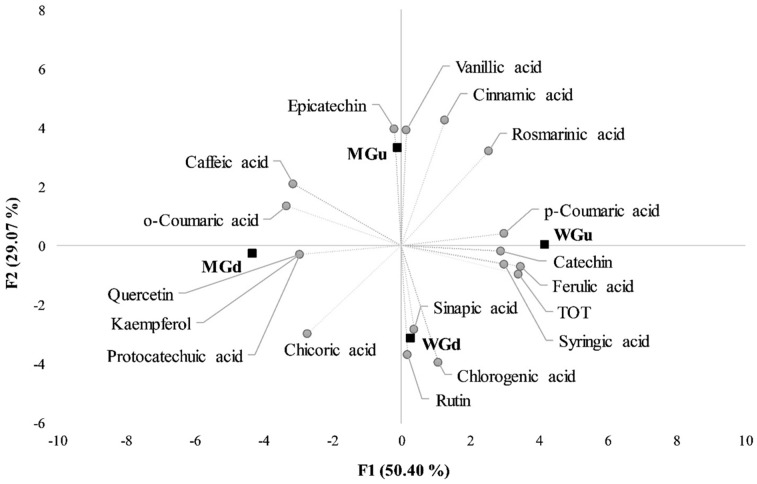
Biplot obtained from the principal component analysis (PCA) of the HPLC-DAD characterisation results. In the figure, MG, micropropagated Genepì; WG, wild Genepì; suffixes u and d refer to undigested and digested samples, respectively; TOT, total concentration of the investigated polyphenolic compounds.

To differentiate the samples and to evaluate which variables influence their location in a bidimensional space, a principal component analysis (PCA) was performed on the autoscaled data of the phenolics pattern (obtained by HPLC) and antioxidant properties, both before and after the digestions (Figure 4).

The two principal components (F1 and F2) explained 79.47% of the total variance, with the first principal component (F1) explaining 50.40% and the second one (F2) 29.07%. The mapping of the samples highlights that the four series of samples (WG and WGd, MG, and MGd) have a clear differentiation among them, as the results are distinct, each located in one of the four quadrants of the biplot. F1 discriminates the samples according to the digestion process, with a significant difference in the loadings related to the phenolics that appear after the digestion (e.g., rutin, quercetin, chicoric acid, and kaempferol). F2 discriminates the samples based on the different initial patterns of the plant extracts. The WG results are highly characterised by the TPC, *p*-coumaric, syringic acid, and catechin, while MG is characterised by vanillic acid and epicatechin.

**Figure 5:**
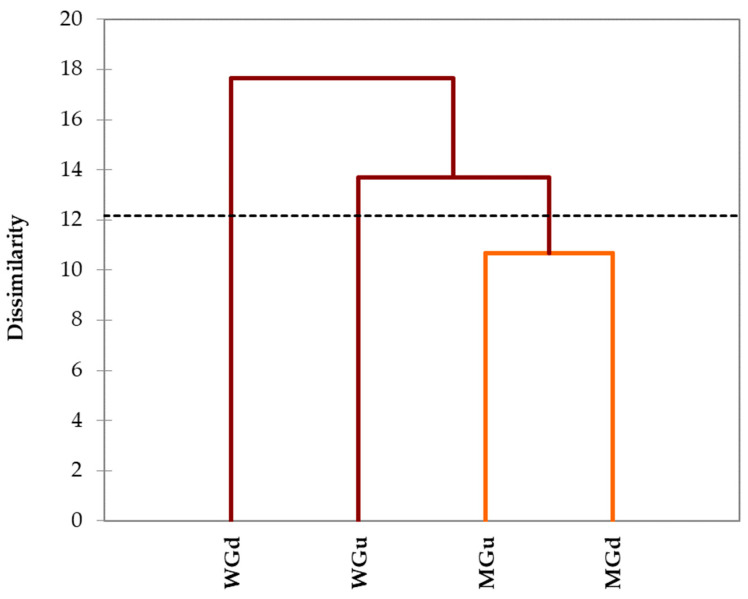
Dissimilarity dendrogram obtained from agglomerative hierarchical clustering of the HPLC-DAD characterisation results. The dash line represents the automatic truncation, leading to two clusters (the first cluster in red and the second in orange).

The same data were processed with a cluster analysis, and the dissimilarity dendrogram obtained is shown in Figure 5.

Clustering allowed the formation of three distinct clusters. In the first cluster was the undigested WG and, in the second one, the digested WG. Conversely, the third class was occupied by both undigested and digested MG. This grouping emphasised that the differences found in the polyphenolic profiles of the digested and undigested MG infusions were not marked. On the contrary, there was a clear distinction between the digested and undigested WG infusions.

## 3. Discussion

*A. eriantha* is characterised by a highly peculiar and characteristic polyphenolic pattern [7]. Environmental factors have a crucial role in the secondary metabolism of plants. These abiotic variables affect not only the accumulation of the compounds but also the metabolic pathways, leading to the synthesis of different types of secondary compounds [34]. A previous study on *A. eriantha* revealed how the location led to a different composition of the nonphenolic secondary metabolites [20]. Several authors also reported how the different altitudes led to a different composition of phenolic compounds [35,36]. The synthesis of secondary metabolites is a defence mechanism of medicinal plants that increases the biosynthesis of phenolics in the presence of low-temperature regimes [37]. Moreover, plants possess a chemical adaption to high-altitude environments, and the impacts of ecological factors on the secondary metabolites are related to their chemical types, structures, and characteristics [34]. The differences in phenolic contents between the WG and MG infusions can also be linked to the different soil compositions and the local microclimatic environments under which the two populations of plants are subjected during growth. In particular, the MG population is located at lower altitudes and under more protected environmental and climate conditions than the WG ones, which, on the contrary, are exposed to higher altitudes and more stressful environmental conditions (e.g., lower temperatures, snow persistence during wintertime, and solar radiation). These variables are already known to positively correlate with phenolic accumulation in several plants that produce these secondary metabolites in response to abiotic stresses [38].

Our results demonstrated that, after digestion, the secondary metabolites profiles change with a significant decrease in the total concentration and the detection of other compounds. These changes could be related to their degradation or conversion to other moieties due to the interaction with other molecules [24]. It is well understood that during digestion, the peculiar physicochemical properties of the gut system along with the presence of enzymes could significantly affect the pattern of both macro- and micro-molecules of an ingested product [39,40,41]. In vitro digestion methodologies could be used as main tool to estimate the impact on the composition of the food matrices, including the secondary metabolites [42]. These changes could only be clarified by studying the interaction between two molecules individually. Nevertheless, it is known that interactions between polyphenols and other compounds during digestion are many and the rearrangements of chemical structures affect essential parameters like bioaccessibility and bioavailability [43].

The results of the in vitro digestion showed that these rearrangements induced the change in total phenolic contents (TPC). Literature survey showed that after digestion, TPC can show both increases and decreases. For example, TPC infusions and extracts of *A. gorgonum* (losna or lasna) and *Hyptis pectinata* (L.) Poit. (commonly named bush mint) [31,44] decreased after GID. Conversely, the increase of TPC after digestion has been described for several aromatic plants and fruit extracts [45,46]. Beyond the already cited metabolic transformations and interactions with other components, pH variations also cause changes in antioxidant activity [45]. Several other variables could be the reasons for this large discrepancy in the scientific evidence regarding the effect of the digestion process conditions. These reasons include the initial phenolics pattern, structural and physical characteristics of the plant matrix used for the extraction, the presence of other molecules in the system under digestion or other analytical aspect related, for instance, to slight variations in the in vitro digestion method used. In their study, Donlao and collaborators reported that the stability of in vitro digestion of green tea infusions’ phenolic compounds is influenced by the roasting and drying temperatures of the tea leaves and, thus, their physical properties [47]. During digestion, chemical compounds are subjected to numerous, and unpredictable reactions and the resultant chemical structures have different abilities to interact with reagents, which affects the obtainable results [48]. It is also worth noting that the Folin–Ciocâlteu reagent can react with other nonphenolic compounds (e.g., vitamins, amino acids, and proteins) in the system, and the final data could be either under- or overestimated [49]. The results of the comparison of the digested infusions with the digestion control (without enzymes) suggest that the increase could be attributed to both the reaction of the assay’s reagent with other components of the mixture or with other compounds produced by several chemical reactions not ascribed to enzymatic activity (e.g., changes in pH and other environmental conditions). Moreover, these drastic changes were not wholly associated with this process. Besides digestion, the losses/increases recorded in the control infusions (without enzymes) of both populations should also be ascribed to other factors that can influence the chemical stability of polyphenols, such as pH changes [24].

From the antioxidant activity point of view, the tests showed that WG infusions have an initial significantly higher antioxidant activity than MG, in agreement with the total phenolics content determined by HPLC. On the contrary, after digestion, a significant decrease occurred, and no significant differences between the two series of samples (WG and MG) were observed. This result could be related to the overall antioxidant activity of a mixture of compound results from that of a single bioactive and its synergic effect [50]. Herbal infusions usually decrease their antioxidant activity after GID [31,44,47,51]. Lima et al. [31] reported similar findings for *A. gorgonum* infusions. At the end of the in vitro digestion process, the concentrations of the investigated polyphenols decreased, and the identification of new compounds after digestion was obtained. Udomwasinakun et al. also described that GID of *A. lactiflora* changed the polyphenolic profiles, recording drastic decreases or disappearance [32]. Moreover, in line with our findings, these authors reported that the different chemical compositions also affected the TPC and antioxidant activity during digestion but maintained a potent antioxidant activity [32].

For all the antioxidant assays, a negative correlation with the TPC was underlined. In fact, with increasing TPC values, the concentration necessary to scavenge the 50% radical potency of ABTS and DPPH increases (highest IC50 values). The scientific literature reports conflicting correlations between antioxidant activity and total phenolic contents, with studies recording positive correlations [52,53,54,55] and others describing very low or no correlations [56,57].

## 4. Materials and Methods

### 4.1. Materials and Reagents

Wild and micropropagated *A. eriantha* (named from now onwards as ‘Genepì’) were collected from the spontaneous meadows of Portella Mountain (2422 m asl, Gran Sasso Monti della Laga National Park) and the flowerbeds of the Giardino Alpino Campo Imperatore (2117 m asl, Gran Sasso Monti della Laga National Park), respectively. Just after collection, the aerial parts were immediately dried at 25 °C for 48 h in a chamber at 15% relative humidity and stored in a glass desiccator jar until processed (final moisture content < 10%).

All reagents and standards were purchased from Sigma-Aldrich (St. Louis, MO, USA). For HPLC-DAD analysis, the standard and reagents were HPLC-grade; reagent-grade quality was used for the spectrophotometric assays.

### 4.2. Genepì Infusions Preparation

The infusions were prepared by using a traditional recipe that guarantees a low concentration of potential toxic compounds naturally extracted from the plant. Briefly, Genepì infusions were prepared from WG and MG by separately adding 0.3 g of dried samples of the aerial parts into 200 mL boiling water and kept at the same temperature for 6 min. Then, the infusions were filtered with a commercial tea filter and left to cool down at room temperature in a hermetically closed glass jar. At least two infusions from both wild and micropropagated plants at different times were prepared.

### 4.3. In Vitro Gastrointestinal Digestion

The in vitro gastrointestinal digestion experiments were carried out following the protocol for liquid meals described in previous works and based on the INFOGEST procedure [30,58,59]. Briefly, 2.5 mL of the infusions were exposed to the gastric and intestinal phases according to the corresponding standard procedure and digesta collected. For the gastric phase, 3.75 mL of simulated gastric fluid (according to [30]), 3 µL of 0.3 M CaCl_2_, 365 µL of water, and 0.8 mL of porcine pepsin stock solution were added to the oral bolus. The pH was adjusted to 2.0 with 1 M HCl, and the mixture incubated at 37 °C in the rotator for 2 h. For the intestinal step, the pH was raised to 7.0 by adding 1 M NaOH after the addition of 5.5 mL of the simulated intestinal fluid (according to [30]), 0.3 M CaCl_2_, and 10 mM bile solution (4.7 mM sodium taurocholate (NaTC) and 5.3 mM sodium glycodeoxycholate (NAGDC)). The solution was incubated for 2 h at 37 °C to mimic the intestinal phase of human digestion. Each digested sample was immediately frozen at −40 °C and freeze-dried for 24 h at 0 °C using a benchtop freeze-dryer (Coolsafe, LaboGene, Lynge, Denmark). Lyophilised samples were then hermetically packed in high-barrier plastic bags and kept at −40 °C until analysis.

For comparison purposes, the initial infusions (not subjected to digestion) were taken and processed as “undigested” samples. Moreover, experiments were also carried out on samples subjected to the same chemical environment of the different digestion steps but in the absence of enzymes (control samples).

### 4.4. Total Phenolic Content and Antioxidant Activity

Both undigested and digested sample infusions of WG and MG were analysed for the total phenolic contents and antioxidant activity using three different methods, namely the DPPH, the ABTS, and FRAP assays.

The total phenolic content was determined by means of Folin–Ciocâlteu reagent following the method described by Singleton & Rossi [60]; gallic acid was used as the reference standard, and results were expressed as mg GA equivalents per dry weight matrix.

The DPPH assay was performed according to the method proposed by Brand-Williams et al. [61]; the ABTS assay was carried out with the method proposed by Gullon et al. [62], and FRAP was assessed by means of the potassium ferricyanide-ferric chloride method described by Oyaizu [63].

For the DPPH and ABTS assays, different sample concentration solutions were assayed to obtain the IC_50_ values (mg mL^−1^ concentration necessary to obtain a scavenge radical activity of 50%).

For the FRAP assay, Trolox (6-hydroxy-2,5,7,8-tetramethylchroman-2-carboxylic acid) was used as the reference standard, and the results were expressed as mg Trolox equivalents per g of dry weight.

Absorbance measurements for the TPC, FRAP, and DPPH methods were carried out by means of the Multiskan™ GO Microplate Spectrophotometer (Thermo Scientific, Waltham, MA, USA).

### 4.5. HPLC-DAD Polyphenolic Profile Analysis

Polyphenolic profiles of the undigested and digested samples of the WG and MG infusions (see Section 4.2) were evaluated by HPLC-DAD analysis using a 1200 series HPLC system (Agilent Technologies, Santa Clara, CA, USA) equipped with a 1200 series DAD (Agilent Technologies). Analytes were identified and quantified using HPLC-grade standards (Sigma-Aldrich, St. Louis, MO, USA), a calibration curve approach (0.1–100 mg L^−1^), and Agilent ChemStation software (Agilent Technologies) following the method previously described [7]. Results were expressed as mg g^−1^ DW.

### 4.6. Statistical Analysis

The data were the means of three different independent experiments, each performed in triplicate. The Student’s *t*-test was applied to compare two means, while, for more than two means, one-way ANOVA was carried out for the comparison. Pearson’s correlation test was applied to evaluate correlations between the TPC and antioxidant activity results. Principal component analysis (PCA) and agglomerative hierarchical clustering (proximity type: dissimilarities applying the Euclidean distance and Ward’s agglomeration method) were also applied to all parameters determined from the samples to determine the similarities and differences among the samples. All statistical tests were carried out using XLSTAT 2016 (Addinsoft, Paris, France).

## 5. Conclusions

For the first time, the present work has provided evidence about the effect of in vitro GID on polyphenolic compounds contained in *A. eriantha* infusions and their antioxidant activities. The results evidenced different behaviours of wild and micropropagated infusions after GID. Our findings represent a valid basis for extensive evaluations on Apennines Genepì infusions and their possible effects on human health. GID has never been applied to infusions or extracts of this species. Thus, all the results obtained expand our knowledge and stimulate new research on the subject. The findings also allowed to shine a light on micropropagated Apennines Genepì. Previous research has already demonstrated that micropropagated Genepì has several potential commercial applications (e.g., essential oils, bioactive compounds, and the low presence of toxic thujones). Therefore, our results add value to micropropagated clones, paving the way for interesting commercial purposes. In addition, having the commercial production of clones available, in return, would reduce the illegal and undiscerning collection from natural stations that threatens this precious species. It will be helpful to increase the number of in vitro studies on the bioavailability of these phenolic substances in contact with various food components to guide the design of functional foods enriched with them. Future studies could be directed towards investigating the best way to store and extract the matrix for the optimal extraction of bioactive compounds. In the meantime, studies on the safety and possible undesirable effects should be carried out to learn more about the extracts themselves.

## Figures and Tables

**Table 1:** Polyphenolic concentration (mg 100 g^−1^ DW) of digested, control (without enzymes), and undigested (initial) infusions of wild and micropropagated Genepì infusions. For wild and micropropagated results in the same row, results followed by different letters were significantly different according to Fisher’s LSD post hoc test (*p* > 0.05).

Compound	Wild Genepì				Micropropagated Genepì			
	Undigested	Control	Digested	LSD	Undigested	Control	Digested	LSD
Caffeic acid	-	-	24.8		67.0 a	69.1 a	75.1 a	9.2
Catechin	172.5 a	71.9 b	11.0 c	42.2	-	-	-	
Chicoric acid	-	-	7.9		-	8.2 b	10.5 a	2.6
Chlorogenic acid	200.4 c	355.2 b	523.6 a	15.8	48.8 b	48.7 b	63.0 a	9.9
Cinnamic acid	45.6 a	13.9 b	10.0 b	1.2	79.5 a	23.7 b	14.6 c	2.5
Epicatechin	-	-	-		89.1 a	15.5 b	4.0 c	2.4
Ferulic acid	163.8 a	156.3 a	132.9 b	6.2	109.4 a	89.7 b	52.4 c	2.6
Kaempferol	-	-	-		-	20.9 b	29.6 a	5.9
*o*-Coumaric acid	33.5 b	39.1 a	41.7 a	10.4	48.0 b	51.5 a	51.9 a	2.9
*p*-Coumaric acid	770.4 a	65.3 b	42.3 c	19.5	107.2 a	45.5 b	-	11.8
Protocatechuic acid	-	-	-		-	3.8 b	12.9 a	4.7
Quercetin	-	-	-		-	74.3 b	127.7 a	35.1
Rosmarinic acid	84.5 a	38.4 b	44.5 b	8	79.2 a	51.4 b	45.2 b	13.8
Rutin	-	-	105		-	-	-	
Sinapic acid	5.5 c	26.9 b	63.8 a	5.7	18.6 a	8.3 b	1.7 c	2.5
Syringic acid	36.0 a	-	6.3 b	7	-	-	-	
Vanillic acid	18.2 a	-	13.6 b	2.7	120.4 a	29.4 b	9.4 c	7.3
TOT	1530.5 a	767.0 c	1027.5 b	77.9	767.1 a	540.2 b	498.0 b	46.5

**Table 2:** Total phenolic content (TPC) and antioxidant activity (ABTS, DPPH, and FRAP) results of the undigested and digested wild and micropropagated Genepì infusions results and Pearson’s correlation coefficients between the different antioxidant activity assays and the total phenolic content.

	**TPC**			**ABTS**			**DPPH**			**FRAP**		
	**(mg GAE g^−1^ dw)**			**(IC_50_)**			**(IC_50_)**			**(mg TE g^−1^)**		
	**U**	**D**	***p*** **-Value**	**U**	**D**	***p*** **-Value**	**U**	**D**	***p*** **-Value**	**U**	**D**	***p*** **-Value**
Wild Genepì	11.48	14.13	***	1.56	4.11	*	2.70	4.64	***	23.62	5.68	**
Micropropagated Genepì	5.29	7.86	***	1.83	4.13	*	3.37	4.88	***	16.26	5.49	**
*p*-value	**	**		***	ns		ns	ns		***	ns	
**Pearson’s correlation coefficient**												
**Assay**				**ABTS**			**DPPH**			**FRAP**		
	**TPC**			0.33			0.12			−0.13		

TPC, total phenolic content; ABTS, antioxidant activity estimated with 2,2′-azino-bis(3-ethylbenzothiazoline-6-sulphonic acid; DPPH, antioxidant activity estimated with 2,2-diphenyl-1-picrylhydrazy; FRAP, ferric-reducing antioxidant power; U, undigested; D, digested. Statistical significance (*p*-value) was calculated according to the Student *t*-test (* *p* < 0.001, ** *p* < 0.01, and *** *p* < 0.05). In columns are reported the statistical significance between the wild and micropropagated undigested/digested results. In rows are reported the statistical significance between the undigested and digested results of wild/micropropagated Genepì. For the Pearson’s correlation coefficients, the positive/negative strength of a correlation was considered: low for ±0.1 < r < ±0.3, moderate for ±0.3 < r < ±0.7, and strong for r > ±0.7; for values of r < ±0.1, the variables were considered not correlated.

## Data Availability

The data that support the findings of this study are available upon request from the corresponding author.
